# A Review of Rigid Polymeric Cellular Foams and Their Greener Tannin-Based Alternatives

**DOI:** 10.3390/polym14193974

**Published:** 2022-09-23

**Authors:** Antonio M. Borrero-López, Vincent Nicolas, Zelie Marie, Alain Celzard, Vanessa Fierro

**Affiliations:** 1 Université de Lorraine, CNRS, IJL, F-88000 Epinal, France or; 2 Institut Universitaire de France (IUF), France

**Keywords:** rigid foams, tannin-based foams, polyurethane foams, phenolic foams

## Abstract

This review focuses on the description of the main processes and materials used for the formulation of rigid polymer foams. Polyurethanes and their derivatives, as well as phenolic systems, are described, and their main components, foaming routes, end of life, and recycling are considered. Due to environmental concerns and the need to find bio-based alternatives for these products, special attention is given to a recent class of polymeric foams: tannin-based foams. In addition to their formulation and foaming procedures, their main structural, thermal, mechanical, and fire resistance properties are described in detail, with emphasis on their advanced applications and recycling routes. These systems have been shown to possess very interesting properties that allow them to be considered as potential substitutes for non-renewable rigid polymeric cellular foams.

## 1. Introduction

It is necessary for industry to adapt to new policies, consumer opinions, and economic demands, and to propose alternatives aimed at the reduction of material costs, the minimization of resource consumption, and the development of more environmentally friendly resources. In order to compensate for the scarcity of petroleum derivatives, raw materials, industrial processes, and production infrastructures are evolving towards the incorporation of bio-based materials and renewable and/or recyclable energy. A large number of political, industrial and academic institutions have therefore embarked on the search for more environmentally friendly energy, chemical, and physical extraction processes.

**Figure 1:**
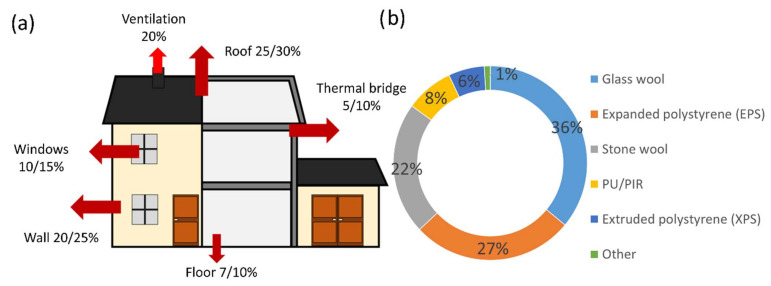
(**a**) Heat losses for a standard building in France [1], (**b**) European insulation market in 2019 [2].

Among these initiatives, this review is focused on recent efforts in the area of the thermal insulation of buildings. Indeed, policies implemented to mitigate the effects of climate change and pollution follow transportation and energy consumption trends with respect to buildings. Numerous incentives to renovate buildings have been launched in order to curb fuel poverty and provide access to healthy, comfortable, and less energy-consuming structures. Thus, new regulations and government subsidies have been implemented, aiming to reduce the energy needs of buildings by improving their bioclimatic design, reducing heat loss (reinforced insulation), promoting solar and internal energy contributions, and installing more efficient systems (heating, mechanical ventilation, etc.). Insulation is therefore a critical point in the design of a new building or the rehabilitation of an old building. The objective is to design an envelope that reduces heat transfer between the interior and the exterior, thus minimizing heat loss. Increasing the overall insulation capacity of the building can be achieved by selecting the appropriate wall, roof, and opening materials, the optimal installation techniques for reducing infiltration losses, and the best design methods for limiting thermal bridges (Figure 1).

Thus, insulating a building involves the use of materials with very low thermal conductivity, as well as the load-bearing structure of the walls. These materials must also meet strict standards with respect to their structural properties (e.g., compressive strength, lack of dimensional shrinkage, fire resistance, durability, etc.) and comply with toxicity regulations. Today, the insulation market includes a wide range of materials. These can be formed of different structures (cellular, fibrous, or loose) and can be composed of various materials (bio-based, synthetic, or inorganic). Each solution has its own facilities and costs of implementation and dismantling that need to be taken into account when making the final choice, depending on the intrinsic performance of the material. Thus, some formulations with a very good thermal resistance have higher installation costs, while less efficient insulators necessitate greater thicknesses, therefore requiring higher volumes to be processed at the end of the product’s life. Glass wool and rock wool are widely used in Europe due to their good performance and ease of installation, as shown in Figure 1. Other solutions also exist, but remain marginal when comparing the volumes used; renewable insulation materials accounted for around 1.4% of the European market in 2019 [2]. Cellulose wadding, straw, and natural fibers such as hemp, flax, wood, and wool can also be mentioned [3,4]. In this respect, the development of a bio-based material with good thermal performance offers an alternative to petrochemical materials.

Inspired by the cellular phenolic and polyurethane materials commonly used in insulation, tannin-based biosourced foams have been designed and produced in recent years at the laboratory scale, with promising performance for insulation and other applications [5,6,7,8,9]. In this review, foams developed for insulation are carefully evaluated through the analysis of the different formulations, foaming methods, and end-of-life processes available for these materials. Finally, special attention is paid to tannin-based foams as an emerging alternative to the use of non-renewable precursors.

## 2. Rigid Cellular Materials

Rigid cellular materials constitute a very broad family. Depending on the application, different kinds of foams can be produced, which possess very different structures covering a wide range of mechanical properties:

- *Metallic foams*, the skeleton of which can, for instance, be made of aluminum, nickel, steel, titanium, copper, etc., offer very good resistance to chemical attacks and high temperature [10].
- *Ceramic foams* based on alumina, silicon carbide, zirconia, etc., are also used for their thermal and chemical resistance.
- *Carbon foams* can be produced from a matrix that is either a polymeric carbon precursor or a support for the deposition of carbon prior to pyrolysis, or can be obtained by exfoliation and compaction of graphite. Their high but narrow porosity, electrical conductivity, and good resistance to high temperatures and fire make them good candidates in applications such as thermal energy storage, electrodes, gas adsorption/desorption, electromagnetic insulation, and fire protection [11].
- *Polymeric foams*, flexible or rigid, are composed of a wide range of macromolecules (polyurethane, polypropylene, polyethylene, polyvinyl chloride, phenol–formaldehyde, melamine–formaldehyde, polyimide, ethylene-vinyl acetate, polystyrene, etc.), and can cover a wide variety of applications depending on the targeted properties and the constraints of price, weight, physical and chemical compatibility.

**Figure 2:**
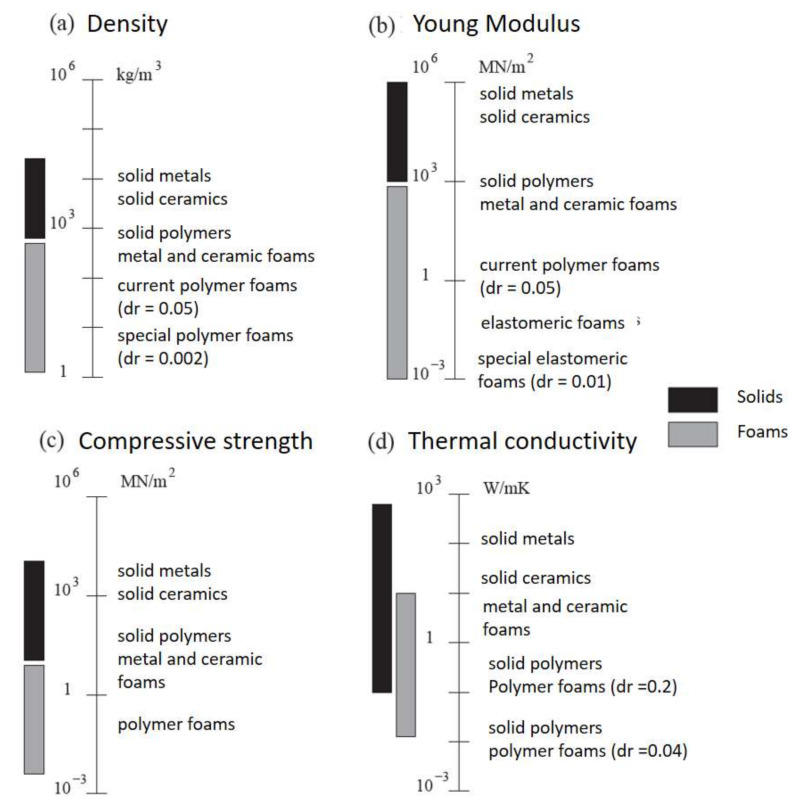
Typical range of values for some properties, i.e., (**a**) density, (**b**) Young Modulus, (**c**) compressive strength and (**d**) thermal conductivity, of foams depending on their nature (adapted from [12]).

As a summary, Figure 2 provides an overview of the usual range of values for the main properties typically used to characterize foams.

The first polymeric cellular materials were designed by incorporating or directly producing gas bubbles within the polymer matrix, resulting in cells. Because of their own polymerization, thermoset foams can in some cases spontaneously produce the gas necessary for the foaming process, whereas when using the thermoplastic method, it is necessary to insert gas bubbles in order to obtain polymeric foams. The final cellular materials can therefore be defined as combining at least two different phases: a porous polymer matrix derived from a liquid reaction mixture or from the cooling of a molten phase, forming a structural skeleton, and a gaseous phase, which is entrapped and forms cavities dispersed therein [13].

The interface between these two phases is called the cell wall, the thickness of which depends on the chemical and physical properties of the matrix, and the proportion of gas introduced [14]. During foam formation, the gas first diffuses into the liquid phase until it forms pockets. Then, the bubbles grow, fed by the continuous diffusion and/or expansion of the gas as the internal pressure and/or temperature in the liquid increases. Finally, the cells can be isolated or merged by creating windows in the walls, an event that opens the pores. Materials are then defined as being “closed cell” when there are no windows in the cell walls. On the contrary, open-cell foams can undergo different degrees of pore opening, from the merging of a few windows to the formation of a structure only based on struts. In this extreme latter case, they are called reticulated foams. The properties of the material, and in particular its structure, depend greatly on the density and distribution of the gas bubbles. Foams can therefore be characterized by considering three different scales of study: microscopic, mesoscopic, and macroscopic.

**Figure 3:**
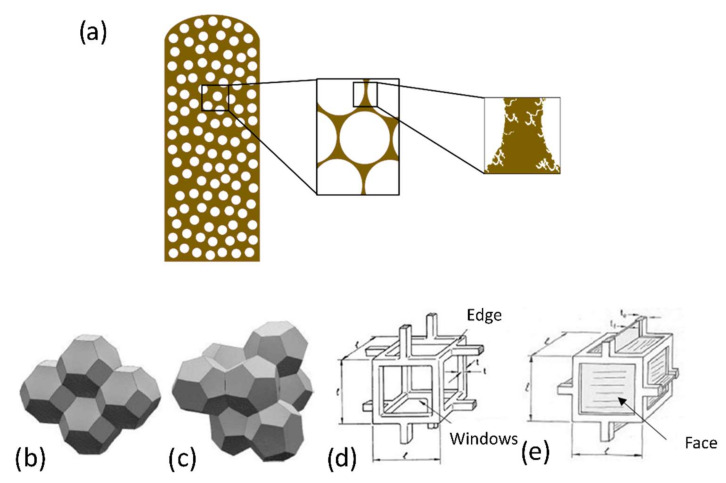
(**a**) Cross-section of a cellular material plane at macroscopic, mesoscopic, and microscopic scales (from left to right). 3D representation of model cell structures according to: (**b**) Kelvin (adapted from [18]); (**c**) Wheaire and Phelan (adapted from [18]); (**d**) Ashby and Gibson (open cells, adapted from [19]); (**e**) Ashby and Gibson (closed cells, adapted from [12]).

- At the *microscopic scale*, pores with a diameter of less than 2 nm, i.e., micropores can be present in the solid walls, in response to mechanical and thermal stresses, and may bear different surface functions depending on the chemical nature of the foam (Figure 3a).
- At the *mesoscopic scale* (2 nm–500 µm), one can observe the arrangement of cells according to the theories of ideal cubic [12], spherical [15], tetrakaidecahedral [16] or mixed [17] structures (Figure 3b–e). At this level, it is possible to determine the size and shape of the cells and to evaluate their orientation, spatial distribution, and possible connectivity by scanning electron microscopy or microtomography (Figure 3a).
- At the *macroscopic scale* (>0.5 mm)*,* the porous characteristics can be distinguished by the naked eye (Figure 3a). Thus, relative density *ρ* can be used to describe the material, defined as the ratio of the apparent (or bulk) density *ρ_a_* (determined by the weight divided by the total volume: solid and cells) to the skeletal density *ρ_s_* (i.e., not considering cells). The porosity, *ϕ* = 1 − *ρ*, is also often referred to as a complement of the relative density. To complete the picture, a cellular material can be characterized by two additional quantities. The tortuosity *θ* is defined as the ratio of the actual length of the flow path when a fluid passes through it to the actual distance between the two ends; and the permeability *k* accounts for the difficulty for that fluid to pass through the medium under consideration when subjected to a pressure gradient. These four parameters are widely used to characterize foams.

### 2.1. Formulations

Throughout history, the discovery of new families of polymeric materials has led to profound changes in the production of goods. As a result, new polymeric materials are lighter, easy and inexpensive to manufacture, and offer a wide variety of shapes and colors. These are employed in industry in order to satisfy the needs of mass production. The global polymer foam market was worth about USD 100 billion in 2015, and was expected to have reached about USD 122 billion by 2021 [20]. More recently, a new report established foam production growth of about 5%/year from 2021 to 2026 [21]. The ubiquity of foams has stimulated studies aiming to improve their chemical, physical, or mechanical properties, while simultaneously achieving greater lightness. Like polymeric resins, foams can be classified into different categories based on:

1. *The chemical mechanisms of polymerization*: polyaddition (production of a polymer chain without by-products); polycondensation (production of a polymer condensate and by-products, such as CO_2_ or H_2_O); cyclotrimerization (production of a polymer composed of cyclic units, obtained from three monomers); ring-opening (production of a polymer by opening the ring from which their monomers are composed); and free radical polymerization (production of a polymer chain by the reaction of radical monomers).
2. *The physical mechanisms for their shaping*: irreversible hardening under the action of heat to obtain thermosetting materials [22], or reversible shaping by softening under the action of heat to shape thermoplastic foams [23], or by crosslinking in elastomers [24].
3. *The mechanical behavior under a light load*: rigid and brittle foams, or flexible and deformable foams, without rupture under load (rubber-like behavior), or semi-rigid foams with an intermediate behavior [25].

To understand the mechanisms involved in the expansion of foams, a literature review was carried out with respect to cellular materials derived from commonly used thermosetting polymers available on the market, as summarized in Table 1. Many thermosetting resins have been developed that form foams when a gas fraction is introduced during synthesis. However, the current market is mainly focused on two large families of materials, which can be characterized on the basis of their main crosslinking agent: (i) foams formed from isocyanates, i.e., comprising N=C=O functional groups; and (ii) foams that instead incorporate phenol derivatives, i.e., composed of an aromatic ring and a hydroxyl group. The former are the main materials used in this sector, and include polyurethanes, polyureas, and polyisocyanurates, while the latter are less widespread and are known as phenolic foams. Polyester foams also exist, but their use is marginal compared to the foams mentions above.

#### 2.1.1. Isocyanate Chemistry

The production of rigid cellular materials, as mentioned above, is largely dominated by polymers derived from isocyanate chemistry, based on the N=C=O functional group. The most emblematic foam in this category is polyurethane foam, from which the other polymers in this family of materials are derived.

##### Polyurethanes

**Figure 4:**
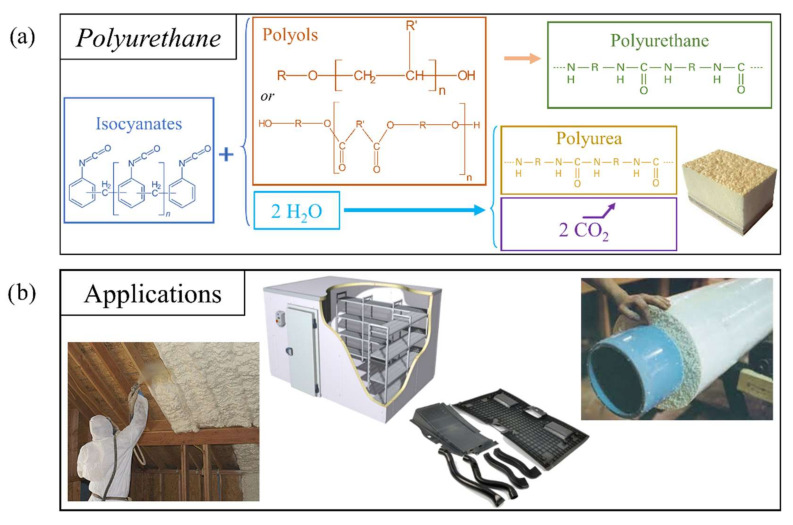
(**a**) Reaction diagram for PUR and PUA systems, containing (from left to right) the isocyanate precursors, some examples of polyols for the formation of PUR, and, in the presence of water, the formation of PUA and the consequent liberation of CO_2_; (**b**) examples of PUR foam applications, such as the thermal insulation of buildings, different polymeric devices, and thermal insulation of pipes.

Polyurethanes (PURs) comprise a family of polymers obtained by polyaddition of urethane units, i.e., the production of urethane units without the formation of by-products during the main reaction, as defined by Carothers [26] (Figure 4a). Nevertheless, careful protocols must be followed if only PURs are being targeted, because through the reaction of isocyanate groups with water or moisture, polyureas can also be formed, as presented in Figure 4a and discussed in the next section.

Developed in the 20th century as a result of the research of Bayer, published in 1937 [27], PURs emerged in German industry as an alternative to hard-to-produce synthetic fibers during World War II, and have become key polymers in the plastics market due to their versatility and their vast number of applications (see Figure 4b) [28,29]. Within this family of PURs, rigid foams account for about 25% of all polyurethanes produced—a market valued at USD 60.5 billion in 2017 [30]. According to a more recent study by Polaris Market Research, growth to USD 92.26 billion is projected for 2026 [31]. The name *polyurethane* does not refer to a unified family of macromolecules like most polymers. In fact, naming conventions set polymer names such that *PolyAAA* is a chain of *AAA* monomer. However, PURs are actually polymeric chains that end in urethane groups, but are not necessarily composed of urethane monomers, as the chains often have other units. The incorporation of additional reagents, with the selected proportions and reaction kinetics, allows the development of myriad polyurethane types, leading to the coverage of a wide range of properties, and thus applications [32]. It is, therefore, difficult to provide an exhaustive list of possible reactions and reactants, but the most notable are mentioned below. However, there are examples of the addition of amines to produce urea units, a possible route for crosslinking through the formation of biurets, carboxylic acids, epoxies, etc. [29]. Modified and hybrid PUR foams based on various proportions of these non-PUR units for mild or advanced modifications, respectively, can be found in the literature [33,34,35].

The polymerization reaction of PUR takes place through a main reaction between isocyanates and polyols and, more generally, compounds rich in oxygen or nitrogen groups such as hydroxyls, amines, amides, acids, epoxides, etc. The main components of foam formulations can be obtained by using foaming agents to allow expansion, catalysts for the different reactions, additives such as surfactants and flame retardants, etc. These different constituents are presented in the following sections.

1. Isocyanates

**Figure 5:**
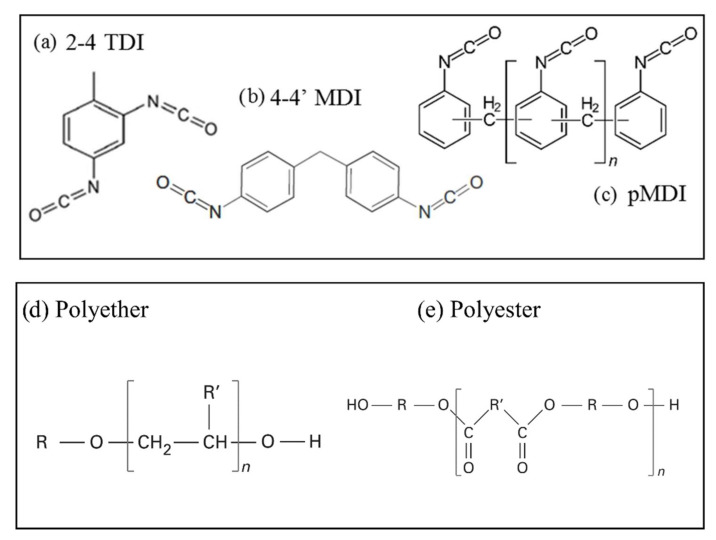
(**a**–**c**) Main isocyanates used in the formulation of PUR. General schematic formulations of main polyols used for PUR production: (**d**) polyether and (**e**) polyester.

Polyisocyanates comprise a family of chemicals that are highly reactive in the presence of a free hydroxyl or amine-rich component, and under appropriate thermal conditions. Thus, they react readily to the addition of alcohols, amines, carboxylic acids, water, etc. Most of the polyisocyanates used in polyurethane synthesis are aromatic compounds, such as toluene diisocyanate (TDI) (Figure 5a), methylene diphenyl diisocyanate (MDI) (Figure 5b), pure monomeric MDI (mMDI), and especially polymeric MDI (pMDI) (Figure 5c), which is the main isocyanate in rigid foams [29,36,37,38,39,40,41,42,43,44,45,46,47,48,49,50,51]. Other isocyanates can be considered for specific uses [29,52,53,54,55].

As mentioned earlier, the synthesis of polyurethanes is based on the reaction between an isocyanate and a polyol generating a chain of urethane units [38]. For foam production, the polyols used in a standard way are often in aqueous medium. When mixed with isocyanates, the main reaction between polyols and isocyanates forms the polymer, while a second reaction between water and isocyanates takes place and produces CO_2_ and urea. As a result, the self-reaction of the isocyanates and the release of CO_2_ become the engine of expansion, thus avoiding the use of flammable or fluorinated foaming agents. In addition, the formed urea units offer improved fire and compression resistance [32].

2. Polyols

The PUR polymerization reaction takes place between any chemical with at least two primary hydroxyl groups and diisocyanates. Polyols are commonly used because of the diversity of their molecules, making it possible to achieve a wide range of properties [29]. The majority of the molecules are oligo-polyols, i.e., polyols with units other than -OH groups in their terminations or in their branches. They generally have a low molecular weight and high functionality, which leads to dense polymerization through crosslinking reactions and thus to rigid matrices [38]. Two main types of polyols can be used for polyurethane formulation: polyethers and polyesters (Figure 5).

Polyether polyols account for 80–90% of the polyols used in the PUR foam market due to their thermal stability, high compressive strength, and the possibility of degradation by hydrolysis of their C-O-C bond (Figure 5d). Their low viscosity and low cost are also advantageous during processing. In addition, their high solubility in many solvents makes it possible to use high-viscosity polyols [29].

Polyesters account for most of the other polyols, acting as aromatic molecules for rigid foams, and aliphatic constituents for other types of polyurethane products [56,57] (Figure 5e), such as polyurethanes alone. Their susceptibility to degradation by hydrolysis and heat has been a huge obstacle in the past, but new environmental and recycling requirements make them ideal new candidates for foam production. Also worth mentioning is the incorporation of halogenated and phosphorated polyols or antimony oxides in polymer chains with flame-retardant properties [29].

3. Additives

The reactions of isocyanates with compounds rich in -OH or -NH groups do not necessarily require a chemical catalyst. However, a catalyst is frequently used when it is desirable to control certain foaming properties (curing, expansion kinetics, etc.), to boost less reactive reactions, or to retard the reaction for specific applications (such as spraying). As an example, the formation of isocyanurates requires a synergistic catalyst composed of a tertiary amine and a metal catalyst [29]. In fact, the two main categories of catalysts used in PUR foaming are: (i) tertiary amines to control the foaming parameters (triethylamine, diazabicycloundecene, etc.); and (ii) metal salts to adjust the reaction kinetics [58]. These can be combined to make the transformation protocol as precise as possible, thus obtaining the optimal desired properties. Other catalysts can also be incorporated, such as buffered amines, or acids like hydrogen chloride, to promote and control reactions to specific products (polyurethane, polyurea, polyisocyanurate, etc.) [58,59,60,61].

On the other hand, foaming agents are also frequently used. The addition of these chemicals to the reaction mixture allows the production of gases that cause the expansion of the polymer matrix. They act by increasing the quantity and pressure of the gas produced. Foaming can occur either by chemical expansion, due to the formation of internal gases, such as the generation of CO_2_ during the reaction between isocyanates and water, or by physical expansion, due to the phase change of a liquid with a low boiling point present in the mixture. Exothermic reactions, or heat input, vaporize the liquid, which is the foaming agent, to generate gas. The most popular foaming agents for rigid polyurethanes include n-pentane and hydrofluorocarbons [62].

Other additives commonly used in PUR formulations include surfactants, which are added to control cell size and degree of opening, flame retardants, and crosslinking agents. In addition, specific applications require the addition of UV stabilizers, dyes, pigments, preservatives, fillers such as fibers to increase matrix strength and dimensional stability, etc.

- Surfactants are generally copolymers chosen to modify the surface tension of the reactive mixture to achieve specific properties, e.g., to help solubilize or disperse a compound, increase the wetting of the mixture in the mold, plasticize the polymer, facilitate the nucleation of gas bubbles, increase the porosity, etc. [63]. To achieve this last objective, a surfactant is commonly added to polymer precursors in order to control the cell size and the type of porosity. Indeed, depending on the properties of the surfactant, the polymer film at the edge of the gas bubbles will be more or less resistant to the gas pressure generated by the bubbles, and will ensure a given dispersion of the latter. The gas bubbles trapped in the polymer mixture grow and create the cells of the matrix. As the material and gas expand, the cell wall becomes thinner and must resist the internal pressure. The ideal surfactant must provide the polymer with enough elasticity to resist deformation during expansion and harden when crosslinking, and likewise during cooling to prevent shrinkage [29]. Therefore, the use of surfactants and the amount added have an impact on the structural, thermal, and mechanical properties of the developed foams [52].
- Flame retardants are chemical reagents added to reduce the thermal degradation and flammability of PUR. Strict regulation with respect to furniture, transportation and construction have spurred research into flame-retardant components, as PUR foams burn easily and can spread incandescent materials. Fire-resistant components work by slowing combustion, incorporating halogens that react with the radicals responsible for increased combustion, and phosphorus that helps to form a carbon barrier and reduce the production of flammable gases. The fire behavior of PURs is the subject of many studies [33,50,64,65,66,67,68,69], including the incorporation of other types of resin in the formulation, such as urea–formaldehyde [70].
- Crosslinking agents and curing agents are chemical compounds that can react with the linear chain of the polymer and link several of its branches. These bonds stiffen the main chain and establish the polymer network with other precursors and additives. Various less common crosslinking reactions may be involved in PUR chemistry, occurring in the final stage of its formation. High temperatures are usually reached in the reactor as a result of the exothermic reactions of urethane formation and CO_2_ generation. These elevated temperatures promote the reaction of isocyanates with other urethane and urea units in the chain or with themselves, forming dimers or trimers. These branches stiffen the matrix and improve the mechanical properties of conventional PUR resins. In addition, PUR foams can be reinforced by adding fibers or other fillers to improve compressive strength [71].

4. Properties and Applications

PUR foams have been used in many technical applications, such as in pressure sensors and bacterial scaffolds, and for oil/water separation [72,73], as well as in insulating materials for buildings, heaters, refrigeration systems, automotive parts, structural components, etc. [29,74,75] (Figure 4b). The diversity of formulations allows a very wide range of mechanical, thermal, water absorption, and fire resistance properties to be obtained. For example, an increase in the amount of surfactant affects polymerization kinetics by increasing the creaming and/or gelling time, which also affects the structural properties by decreasing the density and cell size. These changes have a direct impact on the mechanical properties, resulting in increased compressive strength, and thermal properties, resulting in decreased thermal conductivity [76].

The measured properties and potential applications depend mainly on the formulation and manufacturing conditions (reagent content, additives, water content, temperature, etc.) [76,77,78,79,80,81,82]. For example, rigid foams have a low density (30–58 kg·m^−3^) [83] and a high content of closed cells, which gives them a low thermal conductivity (0.0267–0.034 W m^−1^·K^−1^) [84]. These properties make them good candidates for exterior insulation, heat retention, and, ultimately, energy savings [85]. In addition, polyurethane padding panels based on open-celled foams also offer good acoustic insulation [86] and shock absorption properties [29]. To comply with building regulations, acceptable flame resistance must be guaranteed; therefore, flame retardants can be incorporated to achieve better combustion behavior. Improvements in fire resistance properties have also been achieved using chemical modifications of the polyurethane network, such as via the introduction of urea, isocyanurate, or carbodiimide bonds into the polyurethane chains [87]. Moreover, their cellular organization provides a lightweight and rigid polymeric structure that can also be used for seat frames, vehicle and engine interior components, and decorative structures [88]. Some PUR foams are also ideal for water filtration systems, as they possess a hydrophilic character and an open structure capable of absorbing water [73,89].

##### Polyureas

Polyurea foams (PUAs) have been developed as a less expensive alternative to PUR and polyisocyanurate (PIR) foams. PUAs are cellular materials consisting of a polymeric structure composed of urea units and incorporating optional units of urethanes, isocyanurates, amides, imides, and carbonates [90]. They exist as flexible, semi-flexible and rigid foams, depending on the nature of the cell wall material. PUAs are sprayed or applied in blocks for building insulation or molded into specific and complex shapes in car interiors [91].

**Figure 6:**
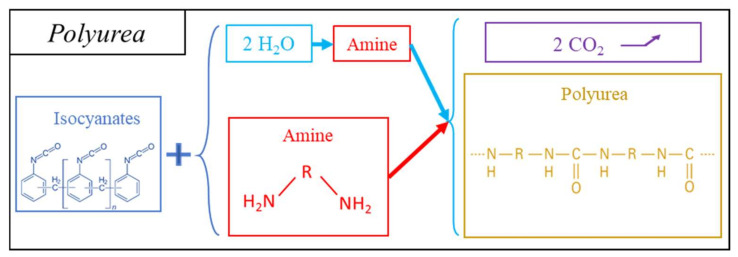
Reaction scheme for the formation of polyurea by contact with water or by direct combination with amines.

PUA foams can also be based on isocyanate chemistry to produce the polymer matrix (Figure 6). Polyureas generally consist of MDI [92,93,94] or pMDI [95,96], which, when coming into contact with water, first produce amines, and then polyureas (see Figure 6). Therefore, to maximize the amount of urea formed by the reaction with isocyanates, some studies incorporate amines directly into the polymer blend, such as aniline [97] or oligomeric diamines [98].

More specifically, the polymerization of urea follows a two-component process through two consecutive reactions. The first is the foaming reaction, which leads to the generation of an unstable carbamic acid that rapidly converts to amine and CO_2_. There has been some research taking advantage of the addition of metal salts such as copper or zinc salts to increase gas generation, thus maximizing expansion [99]. The second step is the polymerization of urea units catalyzed by tertiary amines. Both routes of PUA formation have been described as producing new materials. Water-based formulations have been developed for semi-rigid or rigid building insulation boards and sprayed insulation devices. This is less expensive, because it involves the use of water, which also produces gas for foaming without introducing a foaming agent. However, some foams can be expanded with a fluorocarbon foaming agent [95].

PUA foams have a very low density (~8 kg·m^−3^) and good insulating properties, even though their thermal conductivity is higher than that of PUR foams (0.050 W m^−1^·K^−1^) [93]. As with PUR, some formulations incorporate additives that can also be added to the polymer blend to achieve the desired properties, such as fibrous fillers, which stiffen the polymer network and compensate for poor compression characteristics [94]. There are also surfactants, plasticizers and stabilizers, which provide a specific cell structure in the form of open or closed cells, fine or coarse, or fire retardants added to the mixture to ensure fire resistance and adequate smoke emission.

##### Polyisocyanurates

**Figure 7:**
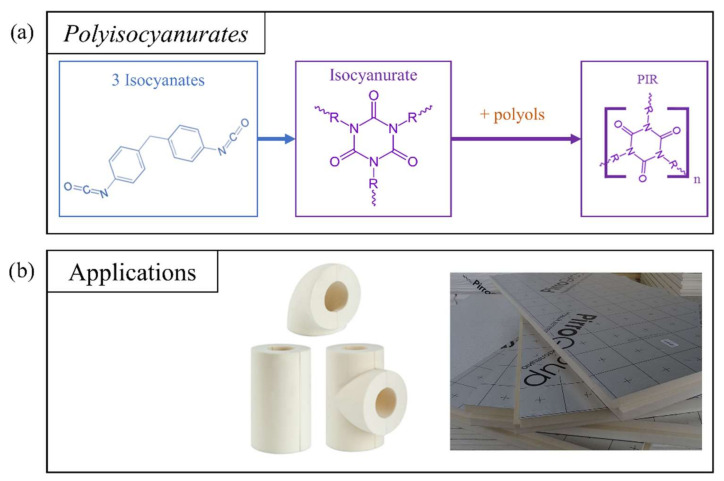
(**a**) Reaction diagram for obtaining isocyanurate from isocyanate and then formulating PIR by adding polyols; (**b**) pipe insulation and insulation panels for covering walls, ceilings, roofs, etc., as examples of PIR foam applications.

Polyisocyanurate, or PIR, foams are formed by a cyclotrimerization reaction, in which three molecules react together in a ring configuration (Figure 7). PIR foams are obtained by reacting high-index isocyanates, where an index is defined as the ratio of the equivalent amount of isocyanate used to the theoretical equivalent amount, multiplied by 100, and in large proportion in a PUR formulation [38,100]. Therefore, PIR foams form a heterogeneous polymer containing an isocyanurate ring linked to polyols through urethane and urea linkages. The foaming agents used for polyurethane chemistry can be applied in the same way to the formulation of PIR foams [101,102]. In addition, four types of catalyst can be used individually or as mixtures: tertiary amines, quaternary ammonium salts, metal salts and potassium-based fatty acids. Other components can be incorporated into PIR formulations, such as surfactants, flame retardants, and fillers.

PIR foams have been designed that retain the advantages of PUR foams while improving their thermal behavior and fire resistance [29]. As a result, PIR foams can be used for construction, thermal insulation, and heating systems, either in panel form or directly foamed on site, [32,103]. Although their fire performance is superior to that of PUR, PIR foams are dangerous when burned, because they are able to release hydrogen cyanide, a compound that prevents the cells from using oxygen [104]. The rigidity and strength of this lightweight material also make it attractive for use in the automotive industry [74,101].

#### 2.1.2. Phenolic-Based Formulations

**Figure 8:**
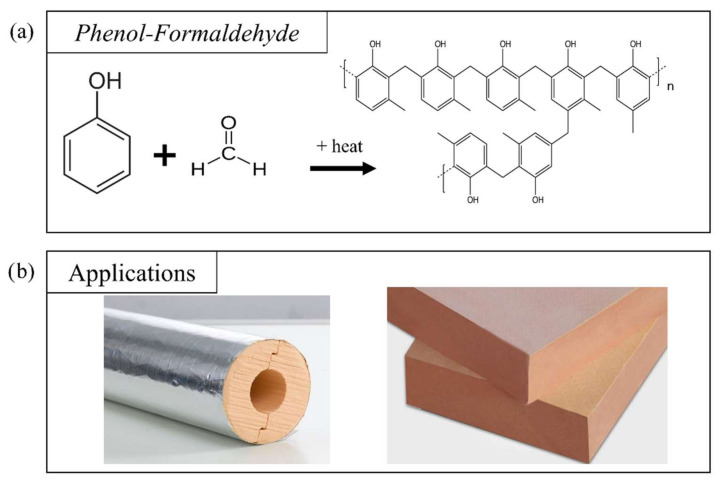
(**a**) General reaction of PF chemistry, showing an example of phenolic precursor, aldehyde crosslinker, and the resulting polymer; (**b**) examples of tubular insulation and panels made of PF foam.

Polyphenolic foams or phenol–formaldehyde (PF) foams refer to a family of foams resulting from the polymerization of phenolic monomers with aldehydes, in particular formaldehyde (Figure 8a) [105,106]. The process can be described in two simultaneous steps: the crosslinking reaction of phenolic compounds and aldehydes; and the foaming of the polymer mixture.

The polycondensation reaction takes place between phenolic compounds and aldehydes. To activate the crosslinking mechanisms, a minimum temperature of 70 °C [107] is typically required, which is achieved either by secondary exothermic reactions, as in the example of tannin–furanic foams, or by external heat input. The thermal conditions applied for each formulation are variable, and depend on the proportion of reactants, catalysts and surfactants used. Some formulation protocols require premixing before being placed in an oven at a temperature between 50 and 140 °C [108,109,110,111], while others subject the material to high temperatures only after expansion for curing [112], which gives the foams different properties.

**Figure 9:**
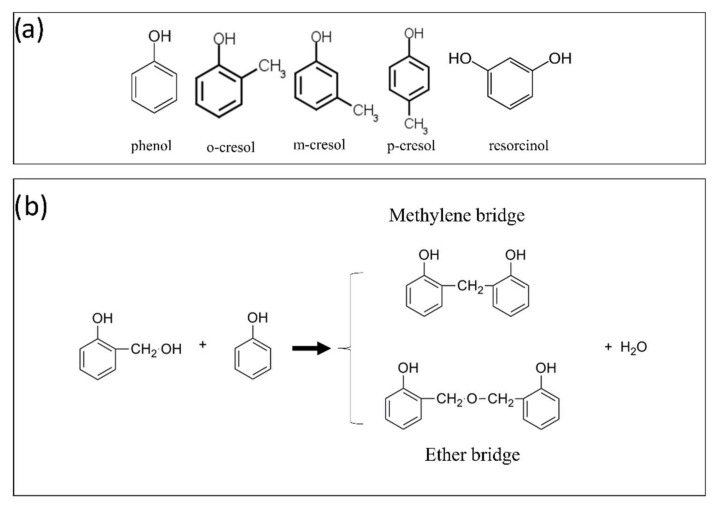
(**a**) Phenol and derived phenolic compounds used in PF foams (based on [113]); (**b**) reaction scheme for the formation of hydroxymethyl phenols (adapted from [113]).

Phenols are aromatic compounds with a hydroxyl function bonded to the ring and have three reactive sites (one in the *para* position and two in the *ortho* positions). As a result, phenols can share three bonds with aldehyde molecules [105], resulting in dense crosslinking, producing the characteristic hardness of these rigid foams. Other phenolic derivatives in which one or more sites have been substituted can also be used for the development of PF foams. Some examples are given below (Figure 9a). These are mainly petrochemical derivatives that can also be found in tars and mineral oils from coal or charcoal [109,113].

The other main reagent of PF foam production is formaldehyde, which is the simplest of the aldehydes. Due to its low price and wide availability, its use is suitable for large industrial volumes. However, other types of aldehydes can be considered to optimize specific properties. Thus, the use of glyoxal, paraformaldehyde, acetaldehyde, or furfural has been reported in the literature [109,113]. Hexamethylenetetramine can also be used for its ability to decompose by the thermal conditions imposed, providing, on the one hand, aldehydes for the PF reaction, and on the other hand, amino constituents that can catalyze it [109].

After mixing, formaldehyde reacts by binding to the C_2_, C_4_ or C_6_ sites of the phenol ring, forming a hydroxymethylphenol. This compound can then polymerize via an ether bond or a methylene bridge, formed by a subsequent reaction with another previously reacted hydroxymethylphenol or a simple phenol, respectively, leading in both cases to the subsequent release of water (Figure 9b). The latter configuration produces bisphenol F, a precursor of polycarbonate plastics. Compared to foams made from other precursors, these generally exhibit less brittleness and fragility, which is the main weakness of PF foams [106]. Therefore, many studies have attempted to improve the mechanical properties of PF foams by adding epoxy resins (EP) or bioresources to the formulation [106,108,110,112]. Another disadvantage that is worth mentioning is that PF foams release acidic vapors over time, which corrode nearby metals, limiting their applications [114]. The phenol–formaldehyde reaction requires the use of an acidic or basic catalyst, characterizing the reaction pathways known as the Novolac and Resole pathways, respectively. Metal salts can also be used to catalyze these two types of reaction [113].

The Novolac route follows a two-step process. In the first step, excess phenol or cresol is mixed with an acid, which acts as a catalyst. Common examples include sulfuric acid, oxalic acid, hydrochloric acid, sulfonic acid, and p-toluenesulfonic acid [111,115,116]. In a second step, the formed Novolac prepolymer can be molded in the presence of formaldehyde at temperatures between 70 and 140 °C. It behaves like a thermoplastic resin that cures slowly, and therefore requires the addition of a hardener to fix the geometry and obtain a thermoset network. This process favors the formation of methylene bonds, although ether groups can also be formed [113] (Figure 9b). Instead, the Resole route is a one-step reaction process, where excess formaldehyde and phenols react in an alkaline synthesis medium (pH around 10). The process begins by mixing phenol and formaldehyde (with the usual formaldehyde/phenol molar ratio being higher than 1:1) with water at room temperature. The hydroxymethylphenol-rich mixture deforms and hardens, with or without a crosslinking agent, after being placed in an oven at the appropriate temperature [113].

PF foams are obtained by means of physical expansion, i.e., by adding a component that is vaporized during the change of state and becomes gaseous. The list of possible compounds includes diisopropyl ether [117], methyl formate [118], diglycidyl ether [108], pentane [111,116], CFC-113 [107], water [107], etc. Other formulations have been investigated by introducing a compound that degrades upon heating and is released into the matrix, producing a gas that drives expansion [109,110,112]. Finally, the use of microwaves has also been considered [119,120].

PF foams are mainly used as insulators in refrigeration and heating installations or in buildings (Figure 8b). Indeed, they possess good thermal conductivity, with values between 0.029 and 0.060 W·m^−1^·K^−1^ for densities between 60 and 130 kg·m^−3^, values slightly higher than those measured for PUR foams [116,117,119,120,121]. Rickle and Denslow also showed the effect of the presence of water in the formulation on the final conductivity, where decreasing amounts of water resulted in a decrease in conductivity [122]. In addition, PF foams have excellent fire resistance [100,108], making them good candidates for use in building insulation. This performance can be further improved by adding phosphate compounds to the formulation [111]. Nevertheless, PFs have been reported to be brittle materials, and need to be strengthened in order to improve their mechanical properties. Several studies have attempted to improve their mechanical performance by adding different fillers, such as fibers [106,116,121,123,124,125], or by chemical modification. In addition, the chemical similarity of epoxy (EP) to that of PF foams allows the ease of reaction and good mechanical properties of both systems to be combined. Thus, Auad et al. obtained a compressive strength between 620 and 2870 kPa, depending on the PF:EP ratio under consideration, in parallel with good expansion [108], while the literature reports values around 138 and 620 kPa [107]. Yang et al. also obtained foams with higher tensile strength and elongation at break by modification with epoxy resins of a mainly PUR backbone [126]. Other authors, on the other hand, focused their work on more environmentally friendly modifications. For example, De Carvalho et al. [105] achieved improved mechanical properties by combining synthetic phenols and lignin-derived phenols, while compromising the characteristic lightness of polymeric foams. In more recent studies, improvements to the properties of PFs have also been reported through the addition of microcrystalline cellulose, clays, graphene, nano-silica or (nano)-lignin [106,127]. Following this strategy, some alternatives to petroleum-based reagents for foam manufacturing are examined in the next section.

#### 2.1.3. Alternatives Incorporating Bio-Based Reagents

To introduce bio-based materials, much work has been performed on replacing petroleum-based reagents. Thus, a wide range of biosourced PUR foams have been developed, which are known in the literature as bio polyurethanes (bPUR). A first route is to add vegetable oils as alternative polyols, while the use of lignin-derived compounds is also very common. Examples include coconut oil, castor oil, linseed oil, palm oil, soybean oil, sunflower oil, evening primrose oil, and polyols derived from lignin [63,83,86,128,129,130,131,132,133,134,135,136,137,138,139]. There are also fillers and fibers of plant origin such as lignin particles [89]. Studies have also been conducted on other materials in the PUR family using alternatives to bio-based polyols. In addition, new materials are emerging through the removal of isocyanates, the mainstay of PUR chemistry, from PUR foams. Thus, isocyanate-free PURs can be developed by reacting polyols with cyclic and bicyclic diamines and carbonates [140,141,142,143,144].

**Figure 10:**
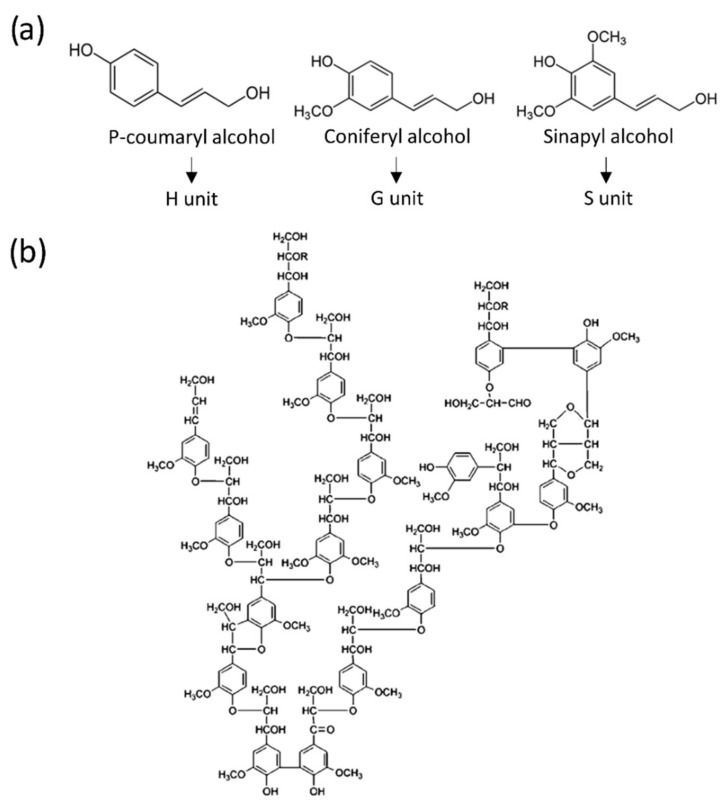
(**a**) Lignin monolignols; (**b**) representation of lignin proposed by Adler (based on [149]). Reprinted from Dobado et al. [149], with permission from Wiley.

Phenolic compound chemistry has also considered renewable sources of phenols. The first method was to synthesize phenolic molecules from bio-based materials. Lignin is a natural three-dimensional polymer that constitutes and provides rigidity and impermeability to the cell wall of plants, of which it is also one of the most abundant components. It is always composed of a combination of the following units: p-hydroxyphenyl, called unit H, derived from coumaryl alcohol; guaiacyl, called unit G, derived from coniferyl alcohol; and syringyl, called unit S, derived from sinapyl alcohol [145] (Figure 10a). Nevertheless, many other phenolic and non-phenolic units are frequently present [146,147]. These three monolignols are derived from phenol, and have been studied to obtain bio-based alternatives to these molecules. Despite the spatial configuration of lignin and the loss of reactivity of the phenolic groups thus extracted, this process has been considered for the chemical valorization of the by-products of the wood and paper industries [107,148]. However, the structure of lignin is complex and difficult to describe as a whole. Its properties depend on the extraction methods used, which partially degrade it. Nevertheless, the model proposed by Adler provides a first approximation of this molecule [149] (Figure 10b). The processes employed in the paper industry and biorefineries aim to separate the carbohydrate components from the lignin, and to valorize this waste, which is very often burned for heat production. However, the most recent research has tended towards finding alternative uses for lignin [147,150,151,152]. Indeed, lignin seems to be promising as a precursor for foams with good thermal properties, low density, and sufficient mechanical strength, and for this reason, there are a number of studies in which phenol is completely replaced by lignin, leading to lignin–formaldehyde foams [153,154]. Lignin has also been considered as a source of phenol and polyol, usually by means of chemical modifications, and can thus be incorporated in PUR or PF blends [155,156].

Among other plant-based alternatives to phenol in PF foams, cardanol has interesting properties, since it is involved in crosslinking with aldehydes. In addition, when it contains phosphorus, it improves fire resistance [157,158]. Finally, tannins, another phenolic extract from plants, are an important area of research as substitutes for PF foams and resins, and are discussed in detail in Section 3.

### 2.2. Foams from Cradle to Grave

The quality of a final foam depends on its thermal, rheological, mechanical, and other properties, which are based on the selection of the precursor, crosslinker and other components. Nonetheless, the selection of an appropriate gas, foaming method and processing method is also necessary to obtain a satisfactory final material. Although very different chemical mechanisms exist, the generation of cellular foams follows the same general steps: mixing of components, processing, expansion, curing, and commissioning of the material before its end of life, as explained in the following sections with reference only to thermoset foams.

#### Foaming Pathways of Thermoset Foams

The cellular structure of such polymeric foams is achieved by a gaseous component whose internal pressure is the driving force for expansion. This gas component, as mentioned earlier, is called the foaming (or blowing) agent, and it depends on the foaming mechanism. In the literature, three types of expansion are generally possible for rigid thermoset foams: chemical expansion using a chemical blowing agent (CBA); physical expansion using a physical blowing agent (PBA); and mechanical expansion (mechanically blown foam, MBF).

##### Physical Foaming

The physical blowing process aims to create gaseous cavities within the polymer by a physical process, hence the name. For example, it can be achieved by introducing a low-boiling liquid PBA into the mixture, which changes phase to become gaseous when the mixture is heated by exothermic reaction or by external input. In contrast, some specific production processes inject the liquid into the mixing head and cause vaporization by depressurizing the liquid at the nozzle [159]. In both cases, the matrix polymerizes and hardens during or shortly after the phase change to trap the gas and stabilize the cell structure.

A promising family was studied and used during the second half of the 20th century: fluorocarbon compounds such as those of the Freon family. They can be distinguished into four subcategories: chlorofluorocarbons (CFCs), hydrochlorofluorocarbons (HCFCs), hydrofluorocarbons (HFCs), and perfluorocarbons (PFCs). The best-known and most widely used fluorocarbon is CFC-11 [160]. Fluorocarbons are carbon chains fully or partially substituted with fluorine and chlorine atoms, and can be handled in liquid form at room temperature. Their low boiling point is often between 20 and 40 °C. This type of blowing agent improves the final properties of foams, providing increased mechanical properties and a closed-cell structure. In addition, they have remarkable thermal properties, with a very low value of thermal conductivity, *λ* [32]. In addition, they have excellent non-corrosive and non-flammable properties. For these reasons, they quickly became the most suitable PBAs for foams in building insulation and refrigeration systems. However, the commercialization of these components was compromised in the late 1980s by the discovery of their involvement in environmental problems. Studies have indeed shown that CFCs and their relatives are responsible for ozone depletion in the stratosphere [161] and contribute to greenhouse effects. As a result, global regulation was adopted by the Montreal Protocol in 1987 [162]. Industries gradually replaced CFCs in their products, until their final ban in 1995, with HCFCs and HFCs, which are less aggressive than CFCs. However, this is a temporary solution, because HCFCs also contribute to the destruction of atmospheric ozone and have a significant global warming impact. Their gradual withdrawal from the market was planned to take place between 1996 and 2030 for developed countries, and 2040 for the rest of the world in the 1992 Copenhagen amendment [163]. The prerogatives of the Montreal Protocol were extended to also include a ban on HFC gases by 2050 by the Kyoto Protocol (COP03) in 1999 [164] and the Kigali agreement of 2016 [165].

Today, PBAs are less frequently used, due to the progressive withdrawal of fluorocarbons from the market. HFCs and PFCs, albeit to a lesser extent because of their chemical incompatibility with polyurethanes, are still used for specific industrial applications. However, companies are looking for alternatives, because these PBAs are more expensive and less efficient than CFCs. Non-fluorinated PBAs have been investigated in the form of hydrocarbon ethers. For example, some research has been conducted with diethyl ether (DE) in a natural PF formulation [9,166,167]. In addition, the use of alkanes such as n-pentane, which has no ozone-depletion potential, and has supplanted fluorinated hydrocarbons in PUR and PF insulating foams, despite its flammability, can also be mentioned [159,168,169]. Its application process and gas content are highly regulated for building insulation. In addition, the spray-applied thickness must be greater to compensate for the difference in thermal properties between alkanes and CFCs. Therefore, research has been conducted on alkanes as PBAs for PUR foam applications to explore new options that meet new economic requirements. Therefore, they must have a low boiling point and be environmentally friendly, non-flammable and cheap. Al-Moameri et al. investigated the cell morphology of PUR foams produced with n-pentane, cyclopentane, isopentane, n-hexane, cyclohexane, a mixture of n-pentane and methylene chloride, and methyl formate in order to estimate the mass transfer coefficients and cell size for each of the considered PBAs by numerical simulation [168,170]. The introduction of CO_2_ as an inexpensive and safe blowing agent has also been investigated recently, with the possibility of creating microcellular epoxy foams with cell densities of about 100 cells/cm^3^ [171]. However, corrugation and surface defects have often been reported due to the use of CO_2_, which is why Breuer et al. tested mixtures of CO_2_ and organic solvents such as acetone, ethyl acetate or ethanol [172].

Finally, the example of physical foaming using microwaves to allow the expansion of phenolic foams can be cited [119,120,173]. The reagents are mixed mechanically to ensure good homogenization and the incorporation of a large fraction of air in the reactive preparation. The latter is subjected to microwave heating between 150 and 250 °C, allowing a more uniform heating of the different constituents than by conduction. However, with this method, foam deformations on the order of 5 to 10 times the initial volume have been obtained. The latest possibilities and main drawbacks of this technique, including the formulation of high-density foams, have been reported elsewhere [174].

##### Chemical Foaming

**Figure 11:**
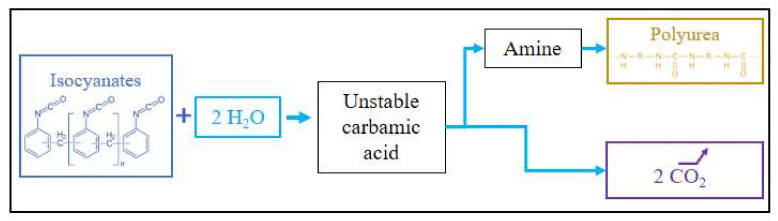
Chemical foaming reaction between isocyanates and water in polyurethane chemistry.

The use of a CBA is the first foaming method historically used in the early days of PUR foam research. This technique involves the production of gas as a by-product of the polymerization reaction or as a side reaction. The most notable example is the production of CO_2_ in PUR foams [32,85]. Water can be introduced into the formulation by the incorporation of free water, or simply by the natural presence of moisture. As shown previously, isocyanates readily react with water to form a carbamic acid (Figure 11). The unstable nature of this component causes it to react instantaneously to produce urea and carbon dioxide. Primarily used for the production of flexible PUR foams, it is also an important means of creating rigid PUR or tannin-based foams [175]. This reaction has been known since the beginning of PUR chemistry. However, investigations into this pathway were slowed down in the 1950s because it was considered to inhibit the main polymerization reaction that produces the PUR chain. Controlling the moisture content of the mixture then became a key parameter in the PUR foam manufacturing process. On the other hand, at the same time, fluorinated PBAs were booming and were used extensively. Nevertheless, their ban prompted companies to reconsider the use of chemical foaming.

Nevertheless, there are also several drawbacks to the reaction of isocyanates with water to produce CO_2_ as CBA, since it requires a larger amount of this component. This is because two units of isocyanate are needed to produce one unit of urea, instead of one single isocyanate producing a unit of urethane. This increases the demand for TDI, and has had a significant impact on the cost of recent products. In addition, the thermal properties of CO_2_ are inferior to those of CFCs, as similar insulating performance would be achieved by increasing the thickness (twice that required with CFCs), which would be achieved with a greater number of reactants [32]. Through chemical foaming, hardening takes place at the same time as the release of the CBA. It stiffens the cell structure during expansion, and it must be controlled to optimize expansion and avoid collapse. The materials produced by this route are therefore slightly different from those physically expanded. They are also generally harder: the heat produced by both reactions without induction of phase change promotes polymerization and hardening, and the cells are generally smaller. These variations affect the structure and properties of the material, and determine the manufacturing process to obtain the desired foam.

##### Mechanical Foaming

A third foaming mechanism has recently been investigated for the removal of hazardous, expensive, and environmentally damaging chemicals from reagent mixtures. In this case, the foaming agent is air, incorporated by mechanical agitation, and trapped in the monomeric solution. Two techniques have been presented for producing MBFs: stiff liquid foam resembling beaten egg whites, for which air is incorporated by whipping with a propeller [176,177]; and foams produced by injecting air into a pressurized nozzle containing the reactive liquid [178]. To optimize the properties of foams, studies have presented ways of combining the effects of two foaming agents. Many examples can be found in the literature on polymer foams blown by a chemical agent and a PBA [168,179,180], as well as studies combining the latter mechanical techniques with chemical routes [176].

### 2.3. End of Life and Recycling

**Figure 12:**
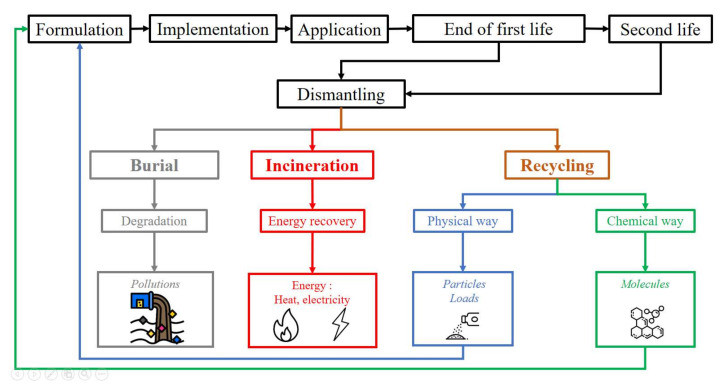
Life cycle of rigid cellular materials.

The polymer foam market was estimated to be worth USD 83.9 billion (25.3 million tons produced) in 2019 for all resins, and is expected to reach USD 134 billion by 2027 [181]. Thus, given this production in recent years, the volume of polymers reaching end of life represents a large amount of waste requiring disposal [182,183,184,185,186]. Such wastes can be sent to traditional generic treatment methods such as incineration or landfill. However, these methods raise environmental concerns regarding groundwater pollution, soil pollution, and greenhouse gas production. The increasing use of polymeric materials brings this issue to the forefront of environmental struggles [187,188,189]. Therefore, alternative methods for managing the end of life of these materials have been studied; in particular, special attention has been paid to the biodegradation mechanism in order to optimize this step, or to the removal of the polymer matrix during recycling for second life applications (Figure 12).

#### 2.3.1. Incineration and Landfill

The two main waste management methods used around the world are incineration and landfill. The incineration process involves the complete oxidation of waste by combustion [85]. The heat produced can be used to contribute to the heating of buildings or to transform this energy into electricity. This combustion also produces ash containing inorganic and metallic elements, smoke, and fine particles. The fumes released by incineration are mainly composed of CO_2_, H_2_O, NO_x_, SO_x_, N_2_, O_2_, HCl, HF, etc., and fine particles, rich in heavy metals, dioxins and furans. Regulations have been imposed requiring these fumes to be filtered before rejecting them into the atmosphere by trapping the particles and dangerous gases. Thus, while this method emits non-recoverable greenhouse gases, which is an obvious drawback, it also produces energy and ash, which can be used as filler in cement, concrete, or asphalt.

Burying waste in landfills is another common method that allows the natural mechanisms of material degradation and decomposition to occur. Microorganisms, bacteria, and enzymes break down polymer chains into smaller molecules that can leach into the soil and water. Most studies on foam degradation have focused on PUR foams [190]. The decomposition of PUR has been studied in order to track the stages of polymer degradation [191]. This study showed that polyester-based PUR was more susceptible to being attacked by aerobic microorganisms compared to polyether-based PUR, due to the more decomposition-resistant chemical structure of polyether. However, PUR resists biodegradation in anaerobic environments and under low-temperature and -humidity conditions [192]. In addition, the stability is highly dependent on the polymer structure and the degree of crosslinking of the network [193]. However, the depolymerization steps are similar between the two types: free isocyanates still trapped in the polymer network are attacked first. Then, the urea and amide bonds are targeted before the degradation of the urethane groups takes place. Finally, the isocyanuric acid rings are the most resistant to microbial activity [194]. Although this is a natural process, possible soil pollution and infiltration into groundwater have led some countries to ban this method. In addition, it wastes land resources for agriculture. It is also worth mentioning that PUR foams degrade faster than other PUR formulations due to their high porosity, which makes them more accessible to potential degradation agents [195]. Further information can be found elsewhere [196,197].

#### 2.3.2. Recycling and Reuse

The previous methods have limited efficiency for waste recovery. Long-term methods are based on physical and chemical recycling, and were compared by Yang et al. [185]. Physical recycling involves treating materials by mechanical or thermal processes. Chemical recycling is the most efficient recycling pathway, but the technology is complex to implement on an industrial scale. Nevertheless, some studies have attempted to obtain compounds by chemical treatment of polyurethanes in order to valorize them for other polymer formulations.

- Physical recycling is based on thermal or mechanical treatments to cause the polymer matrix to break down into smaller segments such as fillers, powders, etc. These solid particles can then be used with a resin to produce composites or to reinforce polymers or other materials such as concrete [198,199]. Gaseous compounds from the thermal recycling of PUR and PIR have also been studied as potential compounds for high value-added uses [200]. They can also be more easily used for further chemical processing, such as hydrolysis, acidolysis or glycolysis [201].
- Chemical recycling is based on the transformation, through chemical reactions, of polymers to produce smaller molecules that can be used for appropriate reactions. Most studies have focused on PUR polymers, one of the main polymers on the market, and are detailed in the literature [185,202,203,204,205,206,207,208,209]. This kind of recovery protocol would be the most comprehensive, giving a second life to polymer waste. However, some steps are complex, requiring a lot of energy, purification, and processing in order to be incorporated into new polymeric structures. Five main reactions have been studied: the alcoholysis, glycolysis, hydrolysis, amine, and phosphate methods [28].
- Alcoholysis consists of the depolymerization of PUR by combining it with a short-chain alcohol and a catalyst to obtain a new polyol, aromatic molecules and amines. When the alcohol used is a glycol, it is called glycolysis.
- Hydrolysis instead involves treating PUR with steam and a metal hydroxide catalyst to produce diamines, polyols, and carbon dioxide.
- The amine process uses a fatty amine to break down the polymer into polyols, aromatic constituents, and amines.
- The phosphate ester process treats the material to produce halogenated components, depending on the initial reactant. The resulting products can be added as flame retardant additives to polymer blends.

Other more recent strategies, such as the catalytic hydrogenation of PUR, have emerged thanks to new catalysts developed recently [210], and acidic processes have also been evaluated [211]. PUR is a very heterogeneous polymer with a variety of formulations, chemical structures, and polymeric structures. Therefore, finding a single route for chemical recovery is extremely complex. Studies on landfill biodegradation have led to the idea of reusing polymers and applying this process in a controlled industrial environment to recover depolymerized chemicals [201]. They focus on biodegradation by genetic and biochemical pathways. The molecules resulting from these depolymerization processes can then be reused as polyols in the formulation of new materials with similar performance to foams produced from virgin compounds [209,212]. The interest in reusing PUR is such that studies comparing reference PUR foams with recycled PUR foams have been carried out, showing minimal changes in mechanical properties [213]. Elastomeric materials based on PUR and ethylene vinyl acetate foams with compositions ranging from 0 to 100% recycled resources have also been evaluated, with 100% recycled formulations exhibiting properties suitable for shoe soles [214]. PUR coatings based on acid-treated PUR waste foams have also been produced, with excellent hydrophobic and mechanical properties [211].

## 3. Tannin–Furanic Foams (TFFs)

Tannin–furanic foams are bio-based formulations in which the initial mixture reacts, self-organizing into a three-dimensional network of polymer chains via crosslinking. The final foam has its final rigid form following a curing step that completes the polymerization.

### 3.1. Formulations

Environmental concerns have led to the search for alternatives to current sources of energy and raw materials. Thus, many studies are trying to develop new polymers based on biosourced materials. The result is the substitution of current non-renewable reagents of existing materials with more environmentally friendly solutions. Various methods of upgrade have emerged to create a wide range of bio-based substitutes [215]. Some improvements have contributed to the creation of new resins, such as tannin–furanic foams. In the 1980s, bio-based alternatives to traditional wood adhesives were presented [216], and these have continued to be studied to the present day [55,150,217]. The polycondensation reaction has been adapted for the design of tannin-based resins and foams that have properties similar to those of products produced from non-renewable resources [167]. While the main research on rigid adhesives and foams is reviewed in the next section, flexible foams and elastomers also open new horizons for the generation of phenol-based polymers [180] and other bio-based structures [152,218,219]. The manufacturing process for tannin-based foams follows the following general formulation: wood tannins, furfuryl alcohol, formaldehyde, catalyst, and foaming agent [167,220].

#### 3.1.1. Tannins

Tannins are phenolic compounds found in different plant tissues such as leaves, fruits, and shells, and especially in the wood and bark of trees [221]. They are involved in the defense mechanisms of plants against parasitic organisms such as insects, fungi, and bacteria [222]. Tannins can be classified into three types of molecules: hydrolyzable, complex, and condensed [223]. The first category refers to a huge proportion of molecules in plants and corresponds to simple phenolic compounds such as gallotannins, gallic and digallic acids, and ellagitannins based on ellagic acid. Tannins from chestnut (*Castanea sativa*) and tara (*Caesalpinia tinctoria*) are among them, and are used in the production of leather goods. However, their low reactivity limits the development of green products based on these molecules. Nevertheless, some studies have attempted to incorporate them as a replacement for phenolic constituents in PF resins or isocyanates in PUR resins [142,224,225].

**Figure 13:**
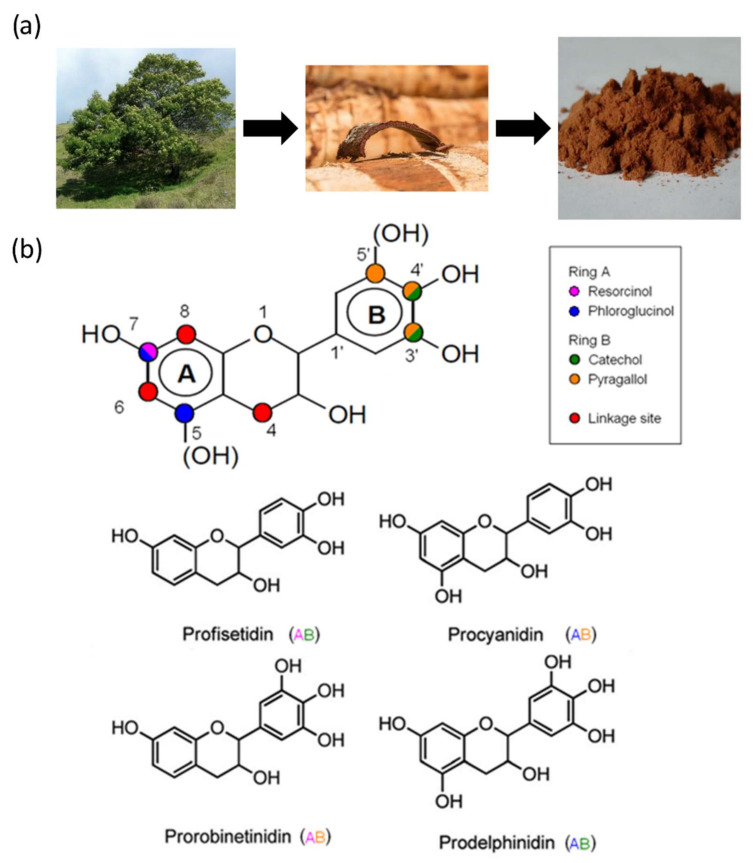
(**a**) Mimosa tree (Acacia mearnsii), mimosa wood after debarking, and tannin powder; (**b**) general structure of flavonoids followed by the main four types of flavonoids (based on [231]). Reproduced from Ref. [231] with permission from the Royal Society of Chemistry.

Condensed tannins are the most studied tannins because they can be recovered from many different trees, including mimosa (*Acacia mearnsii*) (Figure 13a), spruce (*Picea abies*), pine (*Pinus radiata* and *Pinus pinaster*), quebracho (*Schinopsis balansae* and *Schinopsi lorentzii*), and larch (*Larix gmelinii*) [166,226,227,228,229]. Condensed tannins are primarily extracted from barks (Figure 13a) and heartwood, which is achieved by first grinding the original material and then extracting them with solvents, usually hot water with sulfites. The final liquid is filtered and spray dried to concentrate the tannins into a fine brown powder (Figure 13a). Low manufacturing costs, wide availability, and ease of management have enabled the use of these resources in the development of new materials capable of competing with synthetic foams [230].

Condensed tannins are polyflavonoid compounds consisting of 3 to 10 flavonoid units, usually derived from flavan-3-ol and/or flavan-3,4-diol. Each unit is characterized by the combination of an A-ring (resorcinol or phloroglucinol type) and a B-ring (catechol or pyrogallol), as illustrated in Figure 13b. These combinations lead to four main categories of polyflavonoids: procyanidin, prodelphinidin, prorobinetinidin and profisetidin (Figure 13b). Among the most widely used species, mimosa tannins consist of prorobinetinidin units linked together by C_4_-C_6_ bonds. Quebracho tannins, on the other hand, result from profisetidin units linked by C_4_-C_6_ bonds. Pine tannins are mainly composed of procyanidin units, branched by C_4_-C_8_ bonds [232]. Finally, complex tannins are molecules resulting from the bonding of hydrolyzable tannins (ellagitannins) and flavonoid units (flavan-3-ol) [223].

The good reactivity of condensed tannins is a result of their structure, which is characterized by two aromatic rings and nucleophilic OH sites of interest. Previous research has mainly been focused on ring A, which is the most reactive and resorcinol-like, in the case of mimosa tannins, which do indeed resemble the resorcinol used in adhesives. Therefore, the chemistry of these new materials is based on well-known reaction mechanisms. The main reactive mechanism relies on the use of aldehydes to form a polymer under appropriate pH conditions or through catalysts. Under acidic or basic conditions, tannins can self-condense, thus producing more complex polymer networks [233,234]. In addition, tannins also react with added furfuryl alcohol to produce heat for the physical blowing process. Finally, tannins react with hexamine, a compound obtained from the reaction between formaldehyde and ammonia. This component is soluble in water and can be decomposed either in acidic medium into its precursors or in alkaline medium into formaldehyde and trimethylamine. The tannins react with the aldehyde formed and create benzylamine bonds to extend the polymer network [235].

#### 3.1.2. Furfuryl Alcohol

**Figure 14:**
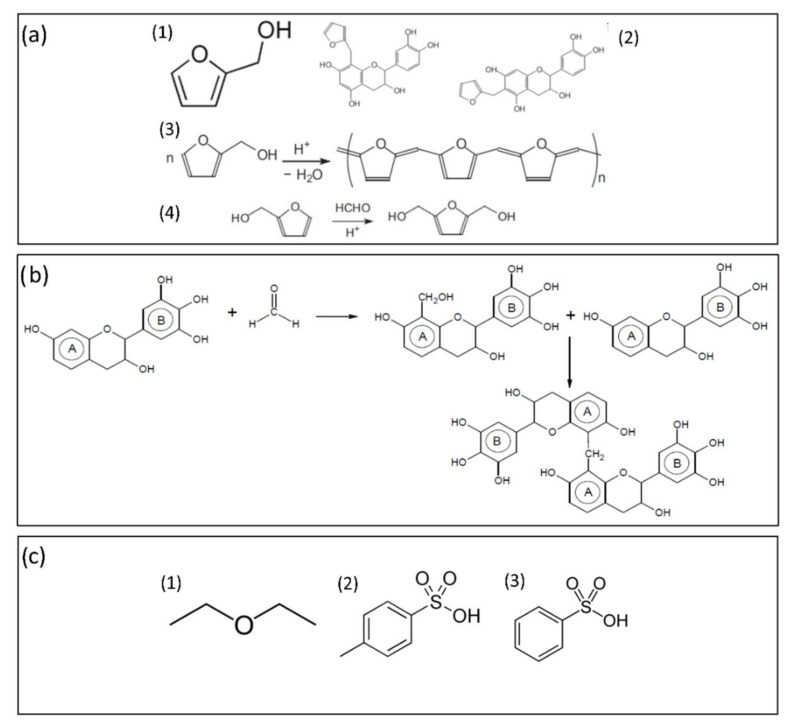
(**a1**) Chemical formula of furfuryl alcohol; (**a2**) products of the reaction of furfuryl alcohol with catechin units of tannin; (**a3**) self-condensation reaction of furfuryl alcohol; (**a4**) reaction between furfuryl alcohol and formaldehyde; (**b**) reaction of formaldehyde with prorobinetinidin (adapted from [11,241]); chemical formula of (**c1**) diethyl ether; (**c2**) para-toluenesulfonic acid; (**c3**) and phenolsulfonic acid.

Furfuryl alcohol (FA) can be derived from furfural, a molecule of great value in the biofuel and green chemistry industries that is obtained by the hydrolysis of C5 sugars contained in hemicelluloses from biomass waste [236,237] (Figure 14(a1)). FA has a good capacity to react with tannins [238] (Figure 14(a2)) and also to self-condense [239,240] (Figure 14(a3)). These two exothermic reactions generate a large amount of heat, triggering the vaporization of the physical foaming agent introduced in the formulation in the case of the physical blowing of the foam. It also participates in the formation of the polymer network. In addition, FA can also be added to the mixture in order to form a highly reactive intermediate with formaldehyde (Figure 14(a4)), which also extends the polymer network.

#### 3.1.3. Formaldehyde

Aldehydes, typically formaldehyde, are added to the mixture as curing agents for the crosslinking reactions. The reactions that take place are similar to those that occur in industrial PF foams between aldehydes and resorcinol. They occur between the aldehyde and ring A, e.g., the resorcinol-type ring in the case of mimosa tannins. It should be noted that under alkaline conditions (pH greater than 10), the reaction with the aldehyde also affects ring B. Following its listing as a human carcinogen by the U.S. National Toxicology Program in 2011 [242], growing concern about the release of formaldehyde has spurred research into possible replacements. Previous work on tannin-based adhesives for wood applications presents several alternatives, considering different aldehydes [243]. For example, formaldehyde-free tannin-based foams have also been studied in the last decade [5,173]. Despite the lower reactivity of alternatives aldehydes, this can be overcome by increasing the amount of FA [175].

#### 3.1.4. Diethyl Ether

Diethyl ether (DE) is used as PBA for “standard” tannin-based foam formulations (Figure 14(**c1**)) due to its low boiling point (34 °C) and absence of reactivity with the other components. Thus, as the temperature of the mixture increases due to the polymerization reactions, DE vaporizes, easily causing the entire mixture to expand.

#### 3.1.5. Para-Toluenesulfonic Acid

A variety of different catalysts can be added to facilitate the desired polymerization reaction under the appropriate pH conditions, depending on the formulation. Two main routes have been investigated: the use of acidic catalysts and the use of alkaline catalysts. Most methods are based on acidic conditions, achieved by the addition of para-toluenesulfonic acid (Figure 14(c2)) or phenolsulfonic acid (Figure 14(c3)) [227,244,245]. The reaction between tannins and formaldehyde is the easiest, but side reactions with isocyanates to produce urethane– and urea–formaldehyde resins have also been considered. For alkaline processes, a sodium hydroxide solution is usually added to the tannin–water–formaldehyde mixture.

#### 3.1.6. Additives

The spectrum of possible applications for these new materials has been broadened through the optimization of the initial formulation and the introduction of new reagents. These additives can be of different types, each of which can be used to improve specific properties. The first example is the use of plasticizers to improve the elasticity of the materials, which are usually rigid, by introducing glycerol [226,246]. The plasticizer promotes the sliding of macromolecular chains relative to each other. Thus, the polymer network gains flexibility and exhibits a more elastic behavior. Conversely, the rigidity of the foam can be enhanced by supplementing the formulation with a filler such as hexamine [235,247], or recent more environmentally friendly alternatives, such as biochar [248,249] or lignin [250]. The addition of isocyanates to create urethane bridges can also be carried out to serve this purpose [180,220,251]. In addition, a good way of combining the effects of physical and chemical expansion in order to obtain a lighter foam is by maximizing cell formation [251]. In addition, surfactants can be included in the mixture to better control the size of the cells, while obtaining a more homogeneous distribution.

### 3.2. Expansion Steps

**Figure 15:**
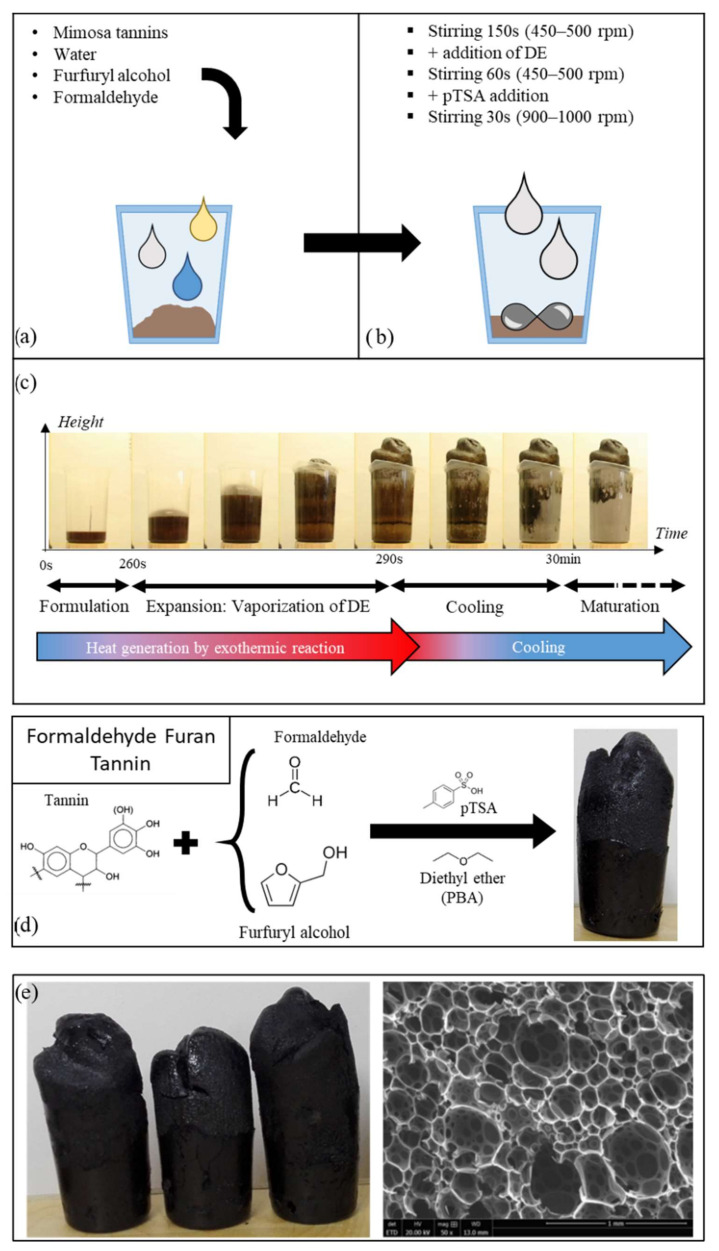
Steps for obtaining TFF foam: (**a**) addition of reagents; (**b**) mixing and addition of PBA and catalyst; (**c**) processing, expansion, and maturation of the material (with an example of time evolution; (**d**) reaction scheme for formulating TFF, (**e**) example of TFs (left) and TFF observed using scanning electron microscopy (×50, right, adapted from Letellier, 2015 [11]).

The expansion of tannin foams follows the same sequence of steps as those presented for other thermoset cellular polymers. Thus, the first step is to mix the polymer fraction without catalyst (tannin, water, formaldehyde, furfuryl alcohol) using a propeller agitator (Figure 15a), resulting in homogeneous and lump-free tannin-based mixtures. The DE is then incorporated into the mixture using a stirrer. Finally, the catalyst is added to the preparation under vigorous stirring; this marks the beginning of the FA polymerization and condensation reaction (Figure 15b). The latter reaction is highly exothermic, and therefore heats the polymer mixture, which maintains constant volume, but exceeds the boiling point of the foaming agent. After a few seconds, the DE changes phase and is vaporized. Then, the internal pressure and gas fraction increase, causing the matrix to expand. The foam reaches its maximum height when the blowing agent has been completely vaporized. Polymerization continues, and the polymer backbone sets and stiffens, forming a 3D polymer network. The material slowly cools following the depletion of the furfuryl alcohol, and completes its curing and drying, which can result in shrinkage in some formulations (Figure 15c). A general overview of the compounds used in the foam formulation and a photo of the final product are shown in Figure 15d.

### 3.3. Physical Properties

#### 3.3.1. Structural Properties

Figure 15e shows the partially open cell structure of a standard TFF. First, this opening of the walls may be a result of the high pressure within the gas bubbles during expansion. When this pressure is too high for the surface tension of the material, windows appear. This phenomenon can also occur at a later stage, during the drying and crosslinking of the polymer matrix, causing the thin walls to shrink, which can ultimately lead to the formation of new windows. The preparation of these lab-made materials in the laboratory is achieved using molds that are taller than they are wide. Expansion is therefore guided so that it only occurs along the vertical axis, creating a slight anisotropy in terms of cell size and shape. Nevertheless, an inherent heterogeneity in rigid tannin–furanic foams has generally been considered, as confirmed in a recent study [252].

**Figure 16:**
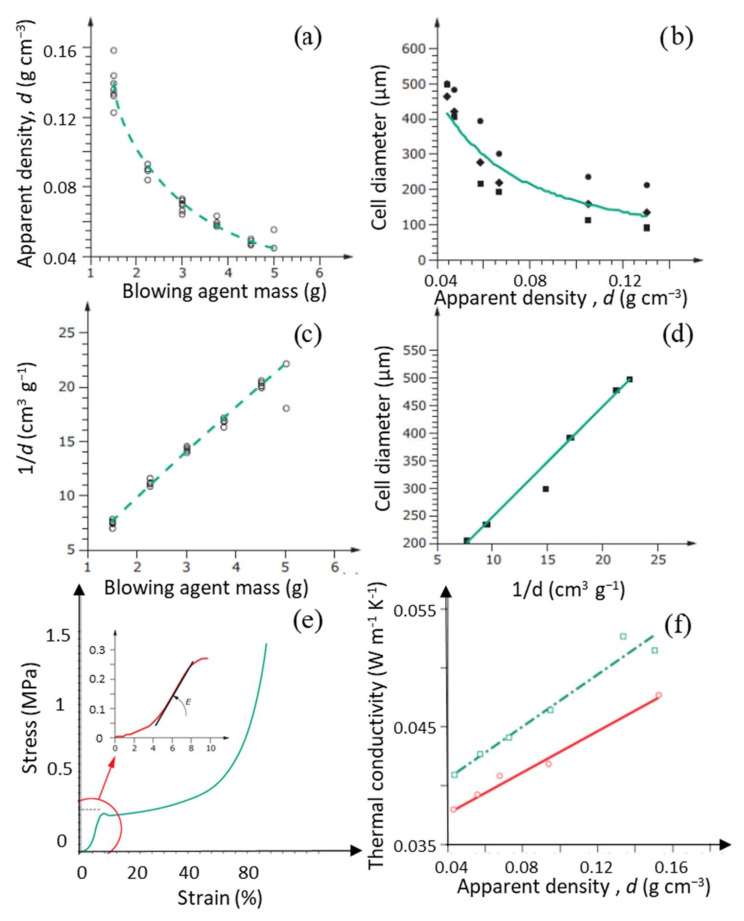
(**a**) Apparent density of TFFs as a function of the mass of introduced PBA; (**b**) cell diameter of TFFs as a function of apparent density; (**c**) inverse of the apparent density of TFFs as a function of the mass of introduced PBA; (**d**) cell diameter of TFFs as a function of apparent density [254]; (**e**) typical compression curve of a TFF (ρ = 0.068kg.m^−3^) (adapted from [7]); (**f**) thermal conductivity of TFFs measured perpendicular to (green dashed line) and along (red solid line) their direction of expansion (adapted from [258]). Figure 16a–d, reprinted from Materials Chemistry and Physics, Zhao et al., Effect of composition and processing parameters on the characteristics of tannin-based rigid foams. Part I: Cell structure, 175–182, Copyright (2010), with permission from Elsevier. Figure 16e, reprinted from Materials Science and Engineering A, Celzard et al., Mechanical properties of tannin-based rigid foams undergoing compression, 4438–4446, Copyright (2010), with permission from Elsevier. Figure 16f, reprinted from Materials and Design, Martinez de Yuso et al., Structure and properties of rigid foams derived from quebracho tannin, 208–212, Copyright (2014), with permission from Elsevier.

A wide range of densities can be covered by adapting the formulation of tannin foams. The choice of foaming method, the proportions of reagents, the use of additives, etc., influence the structure of the foam with respect to cell size, wall thickness, pore opening, and thus the density, which also has implications on the foam’s thermal and mechanical properties. For standard proportions, as presented elsewhere [166], the density of the foam ranges from 0.055 to 0.075 g·cm^−3^, while the density of the solid fraction reaches around 1.59 g·cm^−3^ [220,253,254]. Thus, Zhao et al. implemented TFFs of the same formulation in molds of different diameters (6 and 12 cm) [254]. The materials produced did not show the variations in density or differences in cell shape reported in previous studies [255,256]. It was also highlighted in [254] that limitation of vertical expansion, a recurrent phenomenon for foaming in closed molds or in situ, also disturbed the structure and created anisotropy. The simplest way to control the density is to vary the amount of foaming agent added to the preparation. By introducing different amounts of DE, within safety limits [257], a direct relationship between the mass of DE used and the inverse of the density was demonstrated (Figure 16a,c). Similarly, a relationship was established between the inverse of the bulk density—and thus the mass of DE—and cell size (Figure 16b,d).

However, adjusting the density by varying the other reactants is more complex due to the different chemical reactions that take place during polymerization, making predictions more difficult. For example, increasing the amount of FA may intensify the exothermic self-condensation reaction, generating more heat and facilitating the evaporation of DE. Nevertheless, this also promotes early matrix hardening, thus stabilizing the structure and inhibiting its expansion.

#### 3.3.2. Thermal Properties

Good insulators are materials with low total thermal conductivity, which depends on the contribution of conduction in the solid and gas phases, convection in the cells, and radiation. However, the cell size is generally too small to allow convective motion, so this quantity is neglected [11]. To obtain suitable performance for insulation, the material used must therefore have high porosity, with closed cells, capable of trapping gas of low thermal conductivity. The contribution of the gas fraction depends on the intrinsic conductivity of the gas and the porosity. Many models of porous materials exist for defining the contribution of the solid phase, typically based on the conductivity of the solid fraction and on porosity [12,77]. Finally, the role of radiation must also be taken into account, since it can contribute from 20 to 30% of the total conductivity [11,77].

Like other rigid cellular materials, tannin foams exhibit very good insulating qualities with very a low thermal conductivity (0.032–0.050 W·m^−1^·K^−1^) [8]. However, these results depend on the homogeneity of the structure: in the case of anisotropy, measurements parallel and perpendicular to the expansion axis must be performed, because the conductivities in these directions may differ. In addition, thermal conductivities also depend significantly on the porosity of the material, as mentioned above. In other words, bulk density and total thermal conductivity are correlated, as shown in Figure 16f. Tannin–furanic foams can therefore compete with phenolic or PUR-based foams, and can be considered as a serious alternative for building insulation.

#### 3.3.3. Mechanical Properties

The mechanical behavior of the foam depends on the internal organization of the cells and the degree of polymerization of the matrix. The mechanical behavior is usually characterized by compression both along and perpendicular to the foaming axis in the case of anisotropy. This behavior can be divided into three different phases: the first phase is linear elastic; the second phase, during which stress is maintained while the strain increases significantly, is a serrated plateau; and the third phase corresponds to densification. On the basis of this global behavior, the compressive strength and modulus of elasticity can be obtained as the stress at the end of the linear part and as the slope of the linear part, respectively (Figure 16e). Although the elastic phase of these materials is quite limited, they are capable of absorbing large amounts of mechanical energy by means of irreversible cell wall rupture.

Tannin-based foams are considered rigid materials, i.e., their compressive strength is greater than 0.08 MPa. However, the formulation and addition of hardeners or plasticizers changes the mechanical behavior of the material by altering the bonds between the polymer chains, so that they can be made stiffer or more elastic. With respect to compression, the literature reports values between 0.05 and 1.75 MPa [7,259], which is a good ratio of strength to relative density. These foams are therefore good candidates for use in shock and vibration absorption applications. On the other hand, the removal of formaldehyde from the formulation also increases the elasticity of the material, due to the absence of a rigid 3D polymer network created by the crosslinking agent [5].

#### 3.3.4. Fire Resistance Properties

Tannin foams have very good fire resistance properties, even without the addition of flame retardants [260,261], as the energy input required to start combustion corresponds to severe conditions [220,253,260,261]. For instance, when comparing PF foams and TFFs by applying an incident heat flux of 50 kW·m^−2^, an ignition time of 6s was observed for the former [108], while no ignition was observed for the latter [190]. This unique behavior conferred by tannin allows the derived foams to stand out from other insulation materials, a property that is often a critical point, and is a major vulnerability of PUR foams, in particular. This makes tannins advantageous for applications in sensitive, fire-prone, or public buildings or systems. The numerous studies carried out on TFF foams in which fire resistance properties were evaluated have led to the development of a range of formulations for designing materials with similar or even better properties than the PUR foams they are intended to replace.

### 3.4. Applications and End of Life

The physical and chemical characterizations performed suggest a wide range of possible uses. First, TFFs are lightweight and have low thermal conductivity. Combined with excellent fire resistance, this makes them a good alternative for building insulation. Formulations with open-cell structures have typical acoustic properties for this type of material, even excellent for medium and high frequencies [262]. However, other more sophisticated applications have recently been reported, such as biosubstrates for Raman spectroscopy [263], or the adsorption of different pollutants, as described below. However, one of the main problems related to the use of TFFs for engineering applications is the high friability of these materials. This makes them difficult to handle and is a big obstacle at the industrialization stage. To overcome this disadvantage, they have been studied as core materials of sandwich panels (plywood, medium density fiberboard, wood). The composite materials thus obtained combine the lightness of TFFs and the bending strength of the other elements, without the problem of the friable TFF core crumbling [173,264].

Tannin-based foams are still new products, and are still under development. Therefore, the issue of their dismantling and recycling has not yet been fully explored. Some techniques generally used for cellular materials can be employed, however. Sánchez-Martín et al. developed another application for this material by testing it for wastewater treatment [265]. Tests carried out on dyes, surfactants, and pharmaceutical compounds have demonstrated the good capacity of these foams to depollute [266,267]. This adsorption capacity had already been demonstrated by Tondi et al. for the capture of metal ions (Cu^2+^ and Pb^2+^) from wastewater [268]. This new perspective allows us to consider a method for recycling foams applied as insulation in buildings by transforming them into adsorbents for wastewater remediation. Moreover, other studies have used these materials as precursors to obtain carbon foams by pyrolysis. This step increases the porosity of the materials, in particular by creating micropores. These treatments introduce these foams in areas such as catalysis and absorption [269,270], and pave the way to end-of-life recovery of the foams.

## 4. Conclusions

This review illustrates the wide range of rigid polymeric foam solutions that exist today. However, this mature sector, with well-defined processes and facilities, is still highly dependent on petrochemical feedstocks, which are necessary for the formulation of these polymeric foams. In recent years, some initiatives have attempted to introduce increasing proportions of bio-based materials in order to replace petrochemical reagents. For example, porous materials such as PUR, PIR, PUA and PF foams are playing an important role in this revolution, because their lightweight but robust structure preserves material and energy. Their thermal, mechanical, and acoustic properties make them useful in many applications, the most common being saving energy in the thermal insulation of buildings or industrial systems (coolers, tanks, ducts, etc.). Moreover, these conventional solutions are being progressively challenged by the emergence of new materials such as TFFs, which are positioning themselves as credible alternatives with similar or even better performances than the previous materials, while simultaneously incorporating a higher fraction of renewable raw materials. Although they seem to be promising candidates for replacing PUR or PF formulations, these new products still need to be developed and optimized before reaching the industrialization phase. To conclude this review, Table 2 gathers the main properties of the foams described here.

On the basis of this table, it can be concluded that TFFs have similar or even better properties than those observed for common foams based on non-renewable resources, and thus place them as potential substitutes for many applications.

## Figures and Tables

**Table 1:** Examples of rigid thermosetting cellular materials.

	Poly Addition	Poly Condensation	Cyclo-Trimerization	Ring-Opening	Radical Polymerization
Isocyanates					
Polyurethane PUR (*)	X				
Polyurea PUA (*)	X				
Polyisocyanurate PIR (*)			X		
Polycarbodiimide		X			
Polyimide		X			
Phenolic					
Phenol–formaldehyde PF (*)		X			
Epoxy EP				X	
Tannin–furanic foams TFF (*)		X			
Polyesters					
Unsaturated polyester					X

(*) especially considered in this review article.

**Table 2:** Summary of bulk density, thermal conductivity, compressive strength, and fire resistance properties of the foams reported here.

Foam	Density (g/cm^3^)	Thermal Conductivity (W·m^−1^·K^−1^)	Compressive Strength (MPa)	Fire Resistance	[Refs.]
PUR	0.026–0.058	0.010–0.057	0.45–4.8	Bad	[271,272,273,274,275,276,277,278,279]
PUA	0.008–0.400	0.013–0.050	0.03–2.83	Good	[93,280,281,282,283]
PIR	0.019–0.073	0.032–0.035	0.20–0.32	Good	[29,284,285,286,287,288,289]
PF	0.016–0.314	0.029–0.060	0.07–3.45	Bad	[116,117,119,120,121,220,229,290,291,292]
TFF	0.030–0.200	0.032–0.070	0.05–1.75	Very good	[8,220,253,260,261,293]

## Data Availability

Not applicable.

## Acronyms and Symbols

bPUR	biopolyurethane
CBA	chemical blowing agent
CFC	chlorofluorocarbon
DE	diethyl ether
EP	epoxy
FA	furfuryl alcohol
HCFC	hydrochlorofluorocarbon
HFC	hydrofluorocarbon
MBF	mechanically blown foam
MDI	methylene diphenyl diisocyanate
mMDI	pure monomeric MDI
PBA	physical blowing agent
PF	phenol–formaldehyde
PFC	perfluorocarbon
PIR	polyisocyanurate
pMDI	polymeric MDI
PUA	polyurea
PUR	polyurethane
TDI	toluene diisocyanate
TFF	tannin–furanic foams
*θ*	tortuosity
*k*	permeability
*ρ*	relative density
*ρ_a_*	apparent density
*ρ_s_*	skeletal density (i.e., of the solid fraction)
*ϕ*	porosity
